# Sleep Disturbance from Road Traffic, Railways, Airplanes and from Total Environmental Noise Levels in Montreal

**DOI:** 10.3390/ijerph13080809

**Published:** 2016-08-11

**Authors:** Stéphane Perron, Céline Plante, Martina S. Ragettli, David J. Kaiser, Sophie Goudreau, Audrey Smargiassi

**Affiliations:** 1 Public Health Department of Montreal, Montreal, QC H2L 1M3, Canada; stephane.perron.ccsmtl@ssss.gouv.qc.ca (S.P.); celine.plante.ccsmtl@ssss.gouv.qc.ca (C.P.); david.kaise.ccsmtl@ssss.gouv.qc.ca (D.J.K.); sophie.goudrea.ccsmtl@ssss.gouv.qc.ca (S.G.); 2 Department of Social and Preventive Medicine, School of Public Health, University of Montreal, Montreal, QC H3C 3J7, Canada; 3 Swiss Tropical and Public Health Institute, Basel 4002, Switzerland; Martina.Ragettli@unibas.ch; 4 University of Basel, Basel 4003, Switzerland; 5 Department of Epidemiology, Biostatistics and Occupational Health, McGill University, Montreal, QC H3A 1A2, Canada; 6 National Institute of Public Health Quebec, Montreal, QC H3C 2B9, Canada; 7 Public Health Research Institute, University of Montreal, QC H3C 3J7, Canada; 8 Department of Environmental Health and Occupational Health, School of Public Health, University of Montreal, Montreal, QC H3C 3J7, Canada

**Keywords:** transportation noise, sleep disturbance, land use regression

## Abstract

The objective of our study was to measure the impact of transportation-related noise and total environmental noise on sleep disturbance for the residents of Montreal, Canada. A telephone-based survey on noise-related sleep disturbance among 4336 persons aged 18 years and over was conducted. L_Night_ for each study participant was estimated using a land use regression (LUR) model. Distance of the respondent’s residence to the nearest transportation noise source was also used as an indicator of noise exposure. The proportion of the population whose sleep was disturbed by outdoor environmental noise in the past 4 weeks was 12.4%. The proportion of those affected by road traffic, airplane and railway noise was 4.2%, 1.5% and 1.1%, respectively. We observed an increased prevalence in sleep disturbance for those exposed to both rail and road noise when compared for those exposed to road only. We did not observe an increased prevalence in sleep disturbance for those that were both exposed to road and planes when compared to those exposed to road or planes only. We developed regression models to assess the marginal proportion of sleep disturbance as a function of estimated L_Night_ and distance to transportation noise sources. In our models, sleep disturbance increased with proximity to transportation noise sources (railway, airplane and road traffic) and with increasing L_Night_ values. Our study provides a quantitative estimate of the association between total environmental noise levels estimated using an LUR model and sleep disturbance from transportation noise.

## 1. Introduction

Systematic reviews have shown associations between environmental noise from transportation-related sources and multiple health outcomes, including ischemic heart disease, hypertension, sleep disturbance and annoyance [1,2,3,4]. Recent studies have also shown an association between road traffic noise and diabetes [5], stroke [6,7], mental health [8] and all-cause mortality [7]. Other studies also showed an association between aircraft noise and stroke [9,10].

In several studies, night-time noise has been linked to sleep disturbance [11]. In a systematic review of epidemiological studies, exposure to aircraft noise was associated with multiple indicators of sleep disturbance, including awakenings, decreased slow wave sleep time and the use of sleep medication [12]. Finally, in one experimental study, combined noise exposure from multiple transportation sources (road, railway and air traffic noise) led to more awakenings and arousals than for single noise sources; a similar effect was seen when the number of noise events per unit time increased [13]. To our knowledge, the population health impact of combined train and road traffic noise sources on sleep disturbance has been evaluated in one survey-based study [14]. From a public health perspective, sleep disturbance in and of itself is relevant to health; in addition, it is suspected to be in the causal pathway linking exposure to noise with cardiovascular diseases. Even at very low night-time noise levels, physiological reactions such as an increase in heart rate, blood pressure, catecholamine production and endothelial dysfunction [15,16], body movements and arousals have been measured [3,17]. Night-time activation of the autonomic nervous system has been shown to incompletely habituate to noise [17]. Non-habituating autonomic reactions such as increased heart rate or blood pressure may be important precursors of long-term cardiovascular outcomes [18].

The relationship between environmental noise levels and sleep disturbance has mostly been studied in Europe [19,20]. Sensitivity and exposure to noise may however differ between countries, seasons and individuals. Noise annoyance, an indicator closely linked to sleep disturbance, has been shown to be higher during the summer in European and American cities [21]. Miedema et al. [21] reviewing 42 studies conducted at different times of the year estimated that a 15 °C difference in temperature has about the same effect on noise annoyance as does a 1- to 3-dB difference in noise exposure. Noise sensitivity has been shown to influence sleep disturbance. Indeed, in survey studies and laboratory studies of exposure to transportation noise, subjectively assessed sleep disturbance is higher amongst individuals who report being sensitive to noise [22,23,24,25]. In addition, the link between environmental noise and sleep disturbance has been studied in experimental or quasi-experimental settings, using either direct measurements or noise propagation models that predict noise for a specific source by means of physical rules of noise propagation and attenuation [3,12,24,25]. Recent noise monitoring campaigns in the United States [26] and in three Canadian cities (Halifax [27], Toronto [28] and Montreal [29]) have shown that measured noise levels tend to exceed the 55 dBA L_Day_ and L_Night_ threshold that has been associated with negative health impacts when using propagation noise models [3,4]. In order to accurately quantify the burden of disease associated with environmental noise exposure, it is important to use methods that are based on actual measured noise and control for factors such as sensitivity to noise in the population. In the absence of a noise monitoring system covering the entirety of a large urban area such as Montreal, LUR noise models provide an opportunity to estimate health impacts on the basis of a sample of actual noise measurements. To our knowledge, the effect of environmental noise on sleep disturbance has not been studied using LUR noise models.

The objective of this study was to estimate the prevalence of sleep disturbance associated with exposure to road traffic, railway, airplane and total environmental noise using two exposure metrics—the distance to each transportation noise source; and total environmental noise levels estimated with a land use regression (LUR) noise model amongst residents of the Island of Montreal, Canada. The effect of combinations of noise sources on sleep disturbance was also investigated.

## 2. Methods

### 2.1. Study Population

**Figure 1:**
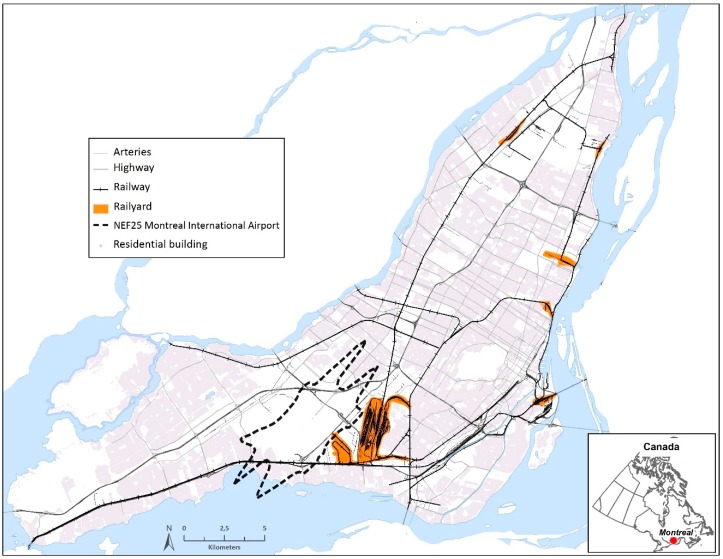
Map of the study area, the island of Montreal, with potential transportation noise sources.

The study population consists of the population of the Island of Montreal—approximately 1.5 million people 18 years or older in 2011 [30]. A significant proportion of the population lives along major roads such as arterials and national highways; some residences are also located in proximity to railway tracks, shunting yards and/or in the vicinity of the Montreal International Airport (Figure 1). Proximity to the airport was defined on the basis of the 2009 estimate of the Noise Exposure Forecast zone 25 (hereafter referred to as NEF25), which circumscribes an area where annoyance is likely to occur because of aircraft noise [29].

### 2.2. Survey and Sleep Disturbance by Noise Data

The data on sleep disturbance used in this study are derived from a telephone-based survey of the health effects of environmental noise carried out in 2014 on the Island of Montreal. A detailed methodology of the survey used for this study has been presented elsewhere [31]. Briefly, survey respondents 18 years of age or older were randomly selected from four pre-defined survey strata (road, rail, aircraft, non-exposed) based on proximity to the noise sources illustrated in Figure 1, in order to ensure sufficient respondents exposed to various transportation noise sources. First, exposure categories were defined on the basis of distance to each transport-related source: living within 100 m of an artery or highway (road); within 150 m of a railway line or main line of a railroad shunting yard (rail); within 1 km of the NEF25 zone of the Montreal International Airport (aircraft); and not within one of the three defined distances to transportation noise sources (non-exposed). The exposure categories were then combined to generate four survey strata: (1) road (road only); (2) rail (rail and road and rail); (3) NEF25 (NEF25, road and NEF25, rail and NEF25, road and rail and NEF25); and (4) non-exposed (an area not directly exposed to transportation noise sources). Noise exposure categories were created by means of ArcGIS 9.2 (ESRI, Redlands, CA, USA) using a digital road network (Addresses Quebec 2013), a railway network (CanMap^®^ Rail V2010.1), and the geo-referenced NEF25 map of the Montreal International Airport [29,32]. We aimed to obtain 4500 respondents (i.e., 1125 respondents for each survey strata).

The survey questionnaire was adapted from the European LARES survey (Large Analysis and Review of European housing and health Status) [33] and included questions on socio-demographic characteristics, self-estimated noise sensitivity and annoyance. The survey was carried out between 10 April 2014 and 20 June 2014. Results on annoyance for the same population are reported in Ragettli et al. 2015 [34].

Sleep disturbance was assessed with two questions. First, a general question on sleep disturbance was phrased as follows: “Was your sleep disturbed by noise in the past 4 weeks?” If the respondent answered yes, the following question was asked: “If yes, what was the source of this noise?” Respondents could choose from the following list of twelve noise sources and/or by an open response item: (1) noise in the neighbourhood (bars, discos, demonstrations); (2) playgrounds, schools; (3) road traffic; (4) airplanes; (5) trains; (6) parking lots; (7) apartments nearby (e.g., voices, music, TV, work, animals); (8) animals or birds (outdoors); (9) shopping centers, industrial or construction zones; (10) Someone using the stairs; (11) ventilation, heating, garbage chute; and (12) noise coming from your own apartment. For the purposes of the present study, four sources of sleep disturbance were retained: individual transportation noise sources—road traffic, railways, airplanes; and total outdoor environmental noise (all sources previously listed except apartments nearby, someone using the stairs, ventilation, heating, garbage chute and noise coming from your own apartment). Sleep disturbance from any of the three transportation sources was also retained as total transportation noise.

Finally, the questionnaire included a question on noise sensitivity phrased as follows: “Please indicate if you are not at all, a little, somewhat, quite a bit or a lot sensitive to noise”. Answers were grouped into two categories: 1. not at all and a little; 2. somewhat, quite a bit or a lot.

### 2.3. Noise Levels and Distance to Noise Sources

A-weighted night-time outdoor total environmental noise levels (L_Night_) for the Island of Montreal were derived at the geographic coordinate of the postal code of each subject using a land use regression model (LUR) developed by Ragettli et al. [34]. On the island of Montreal, each postal code corresponds to a very small geographical area, usually a block-face or a large apartment complex. The median, 5th and 95th percentile of the area of postal codes included in the present study are 7000 m^2^, 1180 m^2^ and 16,018 m^2^, respectively. The LUR model for L_Night_ was developed based on 204 noise samples (175 different geographical sites) collected during a two-week sampling period in the summer of 2010 [29] and in the spring of 2014. The sampling sites were selected in order to be close to residential areas and to cover various transportation noise sources (road traffic, trains and aircraft). At each location, noise levels were measured continuously during at least five days recording two-minute averages. For each site, the equivalent L_Night_ (average) was calculated by integrating all values sampled between 11:00 PM and 6:00 AM. The LUR model was built using a general additive model (GAM) by establishing a statistical relationship between noise measurements and surrounding determinants of the built and natural environments. The model is described in Ragettli et al. [34] and includes predictor variables related to all traffic noise sources: road (length of major roads within 50 m, annual average traffic counts at the nearest road, distance to highway), railway (presence of a railroad shunting yard within 100 m) and air traffic (1 km or less from the NEF25 contour). Characteristics of the built environment known to be associated with noise levels were also added to the model: normalized difference vegetation index (NDVI); geographical sector (West, Downtown, East, Center); residential density; proximity to a bus line; proximity to a river; and presence of commercial land use. Finally, the estimation error of the L_Night_ was assessed for each sampling site using the Root Mean Square Error (RMSE) obtained by leave one out cross validation (LOOCV). The model explained 59% of the spatial variability in L_Night_, and the RMSE was 3.9 dBA.

In addition to L_Night_ for each subject, the distance of the residential six-digit postal code to the nearest transportation noise source (major road, railway, NEF25 contour) was computed using PostGresSQL 9.1 (PostgreSQL Global Development Group, Berkeley, CA, USA). As with Ragettli et al. [34], the distance in meters to the closest major road was divided into six categories (in meters; <50, 51–100, 101–150, 151–200, 201–500, >500), distance in meters to the closest railway (which also includes main railway lines of railroad shunting yards) was divided into five categories (in meters; ≤100, 101–200, 201–500, 500–1000, >1000) [35], distance to the NEF25 contour was categorized into 1000 meter increments (within the NEF contour, >0–1000, 1001–2000, >2000) [36].

To explore the effect on sleep disturbance of being exposed to a single transport-related source compared to multiple sources, we created two additional exposure categories based on the same distance criteria as used to generate the four survey strata (see Section 2.2) but reflecting simultaneous exposure to two sources: road and airplane; and road and rail.

### 2.4. Survey Weights and Statistical Analysis

Data were weighted to generate estimates that are representative of the total adult population of Montreal. First, we computed the sampling weights for each stratum by multiplying the reciprocals of the proportions of respondents by strata and the corresponding proportion of the total population aged 18 and more (obtained from the 2011 Census). These sampling weights were then “raked” so that weighted totals would match census totals for sex and nine classes of age and education (i.e., three classes of education × three classes of age) [37]. Descriptive data and statistical models are all based on weighted data.

In order to quantify the associations between sleep disturbance by source and exposure to noise (categories of distances to source and LUR-estimated L_Night_ as a continuous variable), marginal proportions were computed using the age-sex-education adjusted log-binomial regressions and the “margins” function of STATA 13 (StataCorp, College Station, TX, USA), for the categories of distance (categories described above) and 5-dBA increments in LUR-estimated L_Night_. We also ran a model with all three distance variables. The weighted prevalence of sleep disturbance (i.e., marginal proportions) due to each source (roads, rail, and aircraft) was estimated for categories of distance to the corresponding source in three separate regression models, e.g., proportion of sleep disturbance caused by airplanes according to categories of distance to the NEF25 contour. In addition, the marginal prevalence of sleep disturbance due to road traffic, all transport sources and all environmental sources was computed for each category of LUR-estimated L_Night_ (three models). L_Night_ was used as a continuous variable since the association between L_Night_ and sleep disturbance by environment, transport and roads was linear. We report marginal proportions by increment of 5 dBA.

To explore the effect on sleep disturbance by transportation noise, of being exposed to a single transport-related source, compared to combined sources, we computed a variable classifying the respondents into one of the six following exposure groups: 1-road traffic (≤100 m of major road); 2-aircraft (≤1000 m or inside NEF25 contour); 3-train (≤150 m of a railway); 4-road & aircraft (≤100 m of major road & ≤1000 m or inside NEF25 contour); 5-road & railway (≤100 m of major road & ≤150 m of a railway); and 6-not exposed (>100 m of major road & >150 m of a railway & >1000 m of NEF25 contour). The categories are mutually exclusive and exhaustive. Note that these thresholds correspond to the two first categories of distances to the noise sources described in Section 2.3. Combined exposure to airplanes and railways and combined exposure to all three sources are excluded from the analysis due to an insufficient number of cases. We then computed the marginal proportion of sleep disturbance for each group, based on the regression model relating sleep disturbance by transportation to this variable.

Since noise sensitivity may be an important co-factor in negative health outcomes, we also performed regression models adjusted for sensitivity. Furthermore, regression models were performed with the inclusion of an interaction term between sensitivity and distance or L_Night_. Using these models, we computed marginal proportions of sleep disturbance for categories of distance, and categories of LUR-estimated L_Night_ levels, stratified by sensitivity to noise (not at all/a little and somewhat/quite a bit or a lot). Tests of the significance of interactions terms were performed with the function *testparm* on Stata. STATA version 13.0 (StataCorp, College Station, TX, USA) was used for all statistical analyses.

## 3. Results

In total, 15,697 randomly selected telephone numbers were dialed (of which 21.5% were out of service or not valid) to obtain 4500 respondents. Using the American Association for Public Opinion Research (AAPOR) standards [38], this corresponds to a response rate of 46.8%. After excluding ineligible observations (missing data for age and/or education for generating survey weights), 4336 observations were available for computing the survey weights and carrying out the analyses.

Table 1 presents the characteristics of the unweighted and weighted survey sample. A third of the weighted sample lives within 100 m of a major road. The median of nighttime noise levels L_Night_ is 56.5 and about 60% of the population is exposed to night-time noise levels above 55 dBA according to the LUR model (not shown). Slightly more than half of the respondents are sensitive to noise. Environmental noise is a common cause of sleep disturbance. In this sample, 15.4% of individuals have bad or very bad sleep quality (not shown in table).

**Figure 2:**
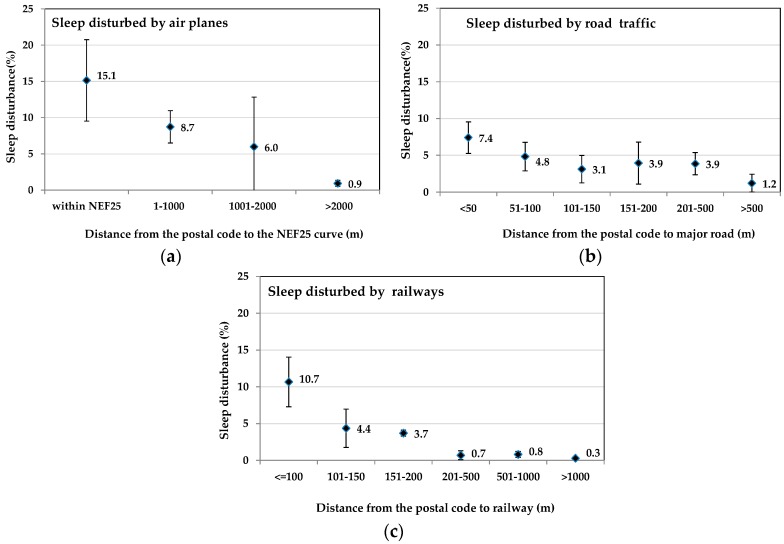
Distance to aircraft NEF25 (**a**), major road (**b**), railways (**c**) and marginal proportions and 95% CI of sleep disturbance by railways in the weighted study sample.

**Figure 3:**
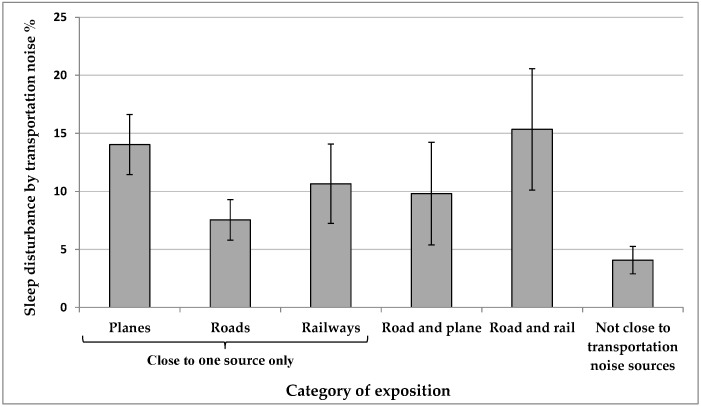
Marginal proportions of sleep disturbance by transportation noise according to proximity to single and combined sources of transportation noise: Airplanes (≤1000 m from NEF25 or in NEF25), roads (≤100 m from an artery or highway) and railways (≤150 m from a railway line or main line of a railroad shunting yard). See Table A16 for the regression model.

**Figure 4:**
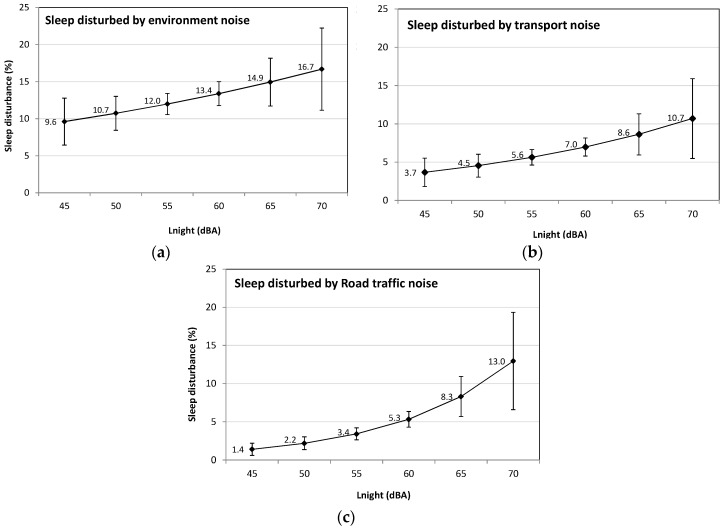
Marginal proportions and 95% CI (confidence interval) of sleep disturbance by environmental noise (**a**); transportation noise (**b**); and road traffic in relation (**c**) to L_Night_ estimated by the LUR noise model in the weighted study sample.

Figure 2, Figure 3 and Figure 4 present the population-weighted prevalence of sleep disturbances according to distance to each transportation noise source. Using log-binomial regression models with the most exposed as the reference categories, negative relationships between sleep disturbance and distance to the noise source are observed for all three transportation sources. The summaries of the regression models are presented in Table A1, Table A2 and Table A3 of the Appendix materials. The strongest effects are observed within 1000 m of the NEF25 contour, 50 m of a major road and 100 m of a railway.

We observed an increase in sleep disturbance due to transportation noise (as defined in Section 2.2) for those exposed to more than one transportation noise source as compared to those not exposed (not close to) to any transportation noise source (Figure 3). Those exposed to both road and rail noise are also more sleep disturbed than those exposed to road noise only or rail noise only but the difference is only statistically significant for road noise. There is also a difference between individuals within 1000 m of the NEF25 contour and those within a 100 m of a major road (Figure 3). Results using distance variables estimated with multivariate models were similar to those obtained with univariate models.

**Figure A1:**
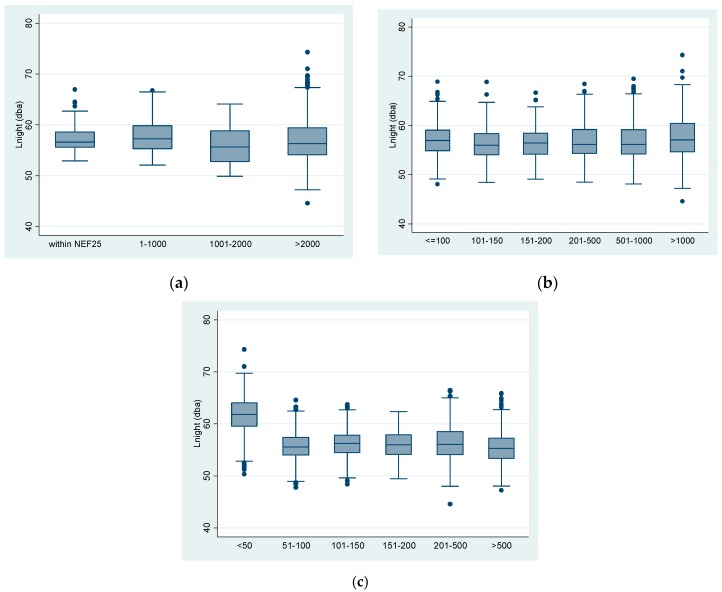
Relation between predicted L_Night_ and distance to: (**a**) NEF25; (**b**) railway; (**c**) major road.

The marginal predictions of sleep disturbance in the total population resulting from models adjusted for sensitivity are very similar to results from unadjusted models (data not shown), although the marginal proportions are higher in sensitive than in the non-sensitive subgroup. Sensitivity to noise does not modify the association between sleep disturbance and proximity to railway sources (Table A4). There appears to be a modification of the association between sleep disturbance and proximity to the airport according to noise sensitivity (Table A6), but the variability of the estimates is large and the interaction term is not statistically significant (*p* = 0.07, Stata *testparm* function). Individuals reporting sensitivity to noise have a higher prevalence of sleep disturbance with increasing proximity to major roads whereas there is no statistical relationship between distance to major roads and sleep disturbance amongst those not sensitive to noise (Table A8). The interaction term for proximity to roads and noise sensitivity is, however not statistically significant (Stata *testparm* function, *p* = 0.34). The marginal predictions of sleep disturbance in the total population resulting from models with interaction terms between noise sensitivity and distance variables are very similar to models unadjusted for noise sensitivity and without interaction terms (Table A5, Table A7 and Table A9). Figure 4 presents prevalence of sleep disturbance in relation to L_Night_ from the LUR noise model. Noise levels derived from the LUR model are correlated with an increase in sleep disturbance caused by environmental, transportation and road traffic noise. At nighttime levels of 60 dBA, 5.3% of individuals have their sleep disturbed by road traffic noise, 7.0% by transportation noise and 13.4% by environmental noise. The relationship between distance to the three single noise sources and L_Night_ levels are presented in Figure A1; there is no relationship between L_Night_ and distance to NEF25 contour or railways.

The marginal predictions of sleep disturbance in the total population (by total environmental noise, transportation and road traffic) resulting from models with L_Night_ adjusted for sensitivity are very similar to the unadjusted predictions (data not shown), although the marginal proportions are higher in the noise sensitive subgroups (Table A10, Table A11, Table A12, Table A13, Table A14 and Table A15). The effect of the interaction between noise sensitivity and L_Night_ is statistically significant for all three transportation sources (Stata *testparm* function, *p* = 0.01). The marginal predictions of sleep disturbance in the total population resulting from models with interaction terms between noise sensitivity and L_night_ are however, very similar to models unadjusted for noise sensitivity and without interaction terms (Table A11, Table A13 and Table A15).

## 4. Discussion

In Montreal, 12.8% of the population reports sleep disturbance related to environmental noise exposure. We observed a strong relationship between sleep disturbance because of road, railway and aircraft noise sources and distance to the respective noise sources. On a population level, road traffic is the most frequent transport-related cause of sleep disturbance reported by respondents in our study. Our data also indicate that certain groups report very high prevalence of sleep disturbance; for example, nearly one in six people living within the NEF25 contour reports sleep disturbance caused by airplanes noise.

There was an association between L_Night_ as modeled with our LUR noise model and sleep disturbance caused by road traffic, transport and outdoor environmental noise. As L_Night_ levels estimated with the LUR model increase, the proportion of individuals that report having their sleep disturbed by environmental noise sources, transport-related sources and by road traffic also increases.

Variability in exposure, measurements methods, confounding factors and populations may explain why results in our study are not transposable to other studies and why there is no consensus on a single dose-response relationship between noise levels and sleep disturbance [39]. In a pooled data analysis of 24 studies by Miedema and Vos (2007), at an estimated exposure level of 60 dBA L_Night_, about 20% of individuals reported sleep disturbance due to road traffic or aircraft noise and 10% due to noise from railways [11]. At equivalent sound levels, sleep disturbance because of road traffic is four times higher in the pooled studies as compared to our study. Some of the differences may be attributable to previous studies’ reliance on numerical propagation models, as compared to the LUR models based on real measurements used in our study. It is possible that under some circumstances, noise levels estimated with propagation models underestimate the true noise levels encountered in the urban environment. LUR models provide estimates for total environmental noise whereas the propagation models estimate source-specific noise levels. A 60 dBA “pure” traffic noise at a level of 60 dBA may be more disturbing than environmental noise at an equivalent level even when close to a road. A study of three cities in Europe comparing LUR models with propagation models showed that for two of the three cities, noise propagation models may underestimate noise levels when compared to LUR models [40]. Although highly correlated, measured noise levels were also found to be higher by 6.2 dBA in Vancouver when compared to propagation models [41]. In our study, the question on sleep disturbance is dichotomous whereas in the pooled analysis by Miedema and Vos, assessment of sleep disturbance varied across studies, which may also influence the results [11]. Finally, almost all studies in the pooled analysis were performed in areas with more temperate climates than Montreal; part of the difference in the relationship between estimated noise exposure and sleep disturbance may stem from better noise insulation in the context of a colder temperature profile [21]. In addition, our survey was performed in the spring when the temperature, in particular night-time temperature, is cooler in Montreal requiring windows to be closed, especially at night. Closed windows strongly attenuate noise exposure from outside sources. In a study conducted in Brussels, Belgium, indoor noise levels in quiet areas were similar to levels in noisy areas because households in noisier areas kept their windows closed, as opposed to those in the quiet areas [42].

In a past Canadian survey, 14% of respondents indicated that road traffic noise often interfered with their ability to sleep in the last year and 26.8% of those living within 30 m of a major road reported that road traffic noise often interfered with sleep [43]. In contrast, our results indicate that 7.4% of individuals living within 50 metres of a major road report having their sleep disturbed in the last month. Discrepancies in the association between proximity to major roads and sleep disturbance between the two studies may be explained by the fact that in Michaud et al.’s study [43], a major road was defined as being either one that has four or more lanes or has a posted speed limit of 80 km an hour and over in both urban and rural contexts. In addition, In Michaud et al.’s (2007) study, distances to the road were self-reported and not defined by GIS [43]. Finally, in our study, major roads were defined as highways or arterial roads, the latter probably emitting less noise.

We did not assess the health status or chronic disease status of the study participants, but have no reason to believe that Montreal’s population is healthier than the populations sampled in the other studies. Although 37.2% of our weighted study sample had a university degree, this is similar to other survey-based studies in European cities such as Malmo, Sweden (44%) [14], Belgrade, Serbia (approximately 34%) [25] and Oslo, Norway (46%) [8]. Although we have no a priori reason to believe that the individual characteristics of our study population account for the differences in the prevalence of sleep disturbance between our study and others, we cannot rule out that certain population characteristics may have contributed to some of the observed differences.

In our study, the overall prevalence of sleep disturbance in the total population was not influenced by noise sensitivity although those sensitive to noise reported higher levels of sleep disturbance, irrespective of the noise source. This is consistent with a Korean and a Norwegian study that reported that sleep disturbance was more pronounced in individuals sensitive to noise [23,24].

We observed more sleep disturbance for those exposed to two transportation noise sources (i.e., rail and road noise sources) compared to those exposed to one source. This is concordant with an experimental study where those exposed to more than one noise source had more sleep fragmentation and worse sleep [13] but not with another study where those exposed were sleep quality decreased equally for those exposed only to road traffic or road traffic and ventilation noise [44]. In another experimental study, individuals reported more annoyance from aircraft noise when exposed to multiple noise sources [45]. We did not detect a trend with regards to aircraft noise in our study. It is however, possible that we did not have the statistical power to detect all trends or that one noise source predominates over the other. The dynamics of two noise sources in a non-experimental setting may also differ according to the noise source; for example, background road traffic noise may attenuate the effects of aircraft peak noise levels. Finally, there may be unmeasured confounding: for example, those exposed to both rail and traffic noise may also be more exposed to noise by heavy vehicles than those exposed only to rail noise. Future studies are needed to better quantify the possible link between the nighttime transportation noise exposure, sleep disturbance and cardiovascular disease.

One of the potential limitations of our study is the cross-sectional design. This design should however, be adequate for measuring outcomes such as sleep disturbance that have a close temporal relationship with the exposure. Our study is limited by the fact that we have no objective measures of sleep disturbance (actigraphy, polysomnography etc.). The relatively low response rate (46.8%), even if the data is weighted to be representative of the population age, sex and education, may compromise the generalizability of our results to the entire population. The use of sleep questionnaires has however, been shown to be a simple, reproducible and inexpensive method for gathering information regarding sleep outcomes [46,47].

There are also limitations related to the use of a LUR model to estimate night noise exposure; our model only explained 59% of the spatial variation of L_Night_ and its error obtained by crossvalidation was 3.9 dBA. In the absence of an independent set of measured noise levels for our study area, we could not assess the performance of the LUR model against actual levels of environmental noise; this would be an important additional step in determining the true error associated with the LUR model. The LUR model cannot isolate a specific noise source whereas propagation models can estimate source-specific noise levels. In addition, since the model integrates noise values between 11:00 PM and 6:00 AM, it does not capture night-time variations; noises of short duration and high intensity (impact noises) and intermittent noise sources are thus less well represented. Recent reviews have stressed that intermittent noise sources have more impact on sleep quality than continuous noise sources [47,48]. This may explain the absence of an observed relationship between L_Night_ and distance to NEF25 contour as well as railways for sleep disturbance and for annoyance as shown in Ragettli et al. (2016) [31]. Our model may better represent road traffic noise, which would explain why a relation was noted between L_Night_ and distance to major roads. The use of the metric termed intermittency ratio (IR) has recently been proposed to predict sleep disturbance [49]. The intermittency ratio expresses the proportion of the total dose of acoustic energy that is attributable to individual noise events above a certain threshold. It is possible that in Montreal, the IR is low in proximity to major roads and higher in proximity to railways and the airport; if this is the case, the IR would be more closely related to distance from these sources than the L_Night_ and enable a better prediction model for sleep disturbance. Further research is needed to develop models that capture the influence of intermittent noise sources such as airplanes and trains. Another limit to be considered is that the use of the postal code to represent the location of respondents’ homes introduces a certain degree of noise exposure misclassification. However, there is no reason to believe that this error is directional and the effect of noise sources on sleep is thereby likely underestimated. Finally, the position of the sleeping quarters relative to the various noise sources, as well as characteristics of the respondents’ homes (insulation, placement of bedrooms, window opening behaviour, etc.) could not be considered. Indeed, a study in Stockholm, Sweden demonstrated that those more exposed to high noise levels kept their windows closed [50]. In addition, there was a better dose-response relationship to noise levels and sleep quality when noise was measured outside bedroom windows instead of the most exposed façade [50].

## 5. Conclusions

Our results clearly demonstrate an association between distance to noise sources and prevalence of sleep disturbance in Montreal. Our study also provides a quantitative estimate of the association between total environmental noise levels estimated using an LUR model and sleep disturbance from transportation noise.

Our study results indicate that sleep disturbance due to transportation noise constitutes a significant public health challenge for an urban area such as the Island of Montreal, in particular amongst individuals living in proximity to major roadways, railways and an international airport. The high proportion of individuals exposed to noise from multiple transportation-related sources and consequent impacts on health and quality of life call for city-wide measures, including zoning restrictions, traffic calming measures and improved soundproofing of private dwellings situated in proximity to noise sources. Future studies are needed to better quantify the possible link between nighttime transportation noise exposure and other health effects such as cardiovascular disease. The health benefits of noise mitigation policies and interventions should also be assessed.

## Figures and Tables

**Table 1:** Weighed characteristics of the study population.

Sample (4336)	Unweighted	Weighted
Age (mean (Standard Deviation (SD)))	53.8 (16.1)	51.0 (15.0)
Female (%)	51.8	53.0
Years lived in the current residence (mean (SD))	13.8 (12.4)	13.0 (10.5)
Educational level (%)		
No diploma or elementary school	8.0	11.0
High school	17.4	25.7
College	26.2	26.0
University degree	48.3	37.2
Exposed to airplane noise: ≤1 km from NEF25 curve (%)	24.4	4.8
Exposed to road traffic noise: ≤100 m from a major road (%)	39.1	33.4
Exposed to railway noise: ≤150 m from a railway (%)	19.1	7.3
Night-time noise level (dBA)		
Min	44.6	44.6
Median	56.1	56.5
Max	69.7	69.7
Sensitivity to noise (%)		
Somewhat/Quite a bit/a lot vs. Not at all/A little	53.8	52.2
Sleep disturbed in the past 4 weeks (%) by		
Environmental noise	15.2	12.4
Total transportation noise	8.9	6.1
Traffic noise	4.5	4.2
Railway noise	2.2	1.1
Aircraft noise	3.4	1.5

## Appendix

**Table A1:** Log-binomial regression models for sleep disturbance due to airplanes in relation to the distance to the NEF25 contour of the Montreal International Airport. All variables are in a same model.

Variable	PPR	SE	*p*-Value	(95% CI)
Distance to NEF25 (m)				
within NEF25	1			
1–1000	0.525	0.136	0.013	(0.315, 0.874)
1001–2000	0.346	0.229	0.108	(0.095, 1.263)
>2000	0.051	0.015	0.000	(0.028, 0.092)
Age				
18–29	1			
30–39	8.030	8.633	0.053	(0.976, 66.087)
40–49	10.507	9.871	0.012	(1.665, 66.285)
50–59	8.828	7.997	0.016	(1.495, 52.141)
60–69	11.741	10.773	0.007	(1.943, 70.951)
70–79	5.596	5.188	0.063	(0.909, 34.457)
≥80	4.723	5.179	0.157	(0.550, 40.547)
Education				
No diploma	1			
High school	3.754	2.552	0.052	(0.990, 14.231)
CEGEP	2.843	1.872	0.113	(0.782, 10.340)
University degree	2.055	1.279	0.247	(0.607, 6.963)
Sex				
Men	1			
Women	1.268	0.403	0.454	(0.681, 2.363)
Constant	0.008	0.008	0.000	(0.001, 0.064)

CEGEP: equivalent to technical school education and is the equivalent of the last year of high school in American colleges and the first year of university. PPR: prevalence proportion ratio. CI: confidence interval.

**Table A2:** Log-binomial regression models for sleep disturbance due to road traffic in relation to the distance to major roads. All variables are in a same model.

Variable	PPR	SE	*p*-Value	(95% CI)
Distance to a major road (m)				
>50	1			
51–100	0.684	0.173	0.132	(0.417, 1.122)
101–150	0.418	0.140	0.009	(0.217, 0.807)
151–200	0.532	0.210	0.111	(0.245, 1.155)
201–500	0.521	0.128	0.008	(0.322, 0.842)
>501	0.160	0.085	0.001	(0.056, 0.455)
Age				
18–29	1			
30–39	1.668	0.880	0.333	(0.592, 4.693)
40–49	1.813	0.923	0.243	(0.668, 4.919)
50–59	2.685	1.358	0.051	(0.996, 7.238)
60–69	1.793	0.980	0.286	(0.614, 5.233)
70–79	1.002	0.644	0.998	(0.284, 3.530)
≥80	1.620	1.204	0.516	(0.377, 6.954)
Education				
No diploma	1			
High school	0.431	0.192	0.059	(0.180, 1.032)
CEGEP	0.851	0.338	0.684	(0.390, 1.854)
University degree	0.723	0.271	0.387	(0.346, 1.508)
Sex				
Men	1			
Women	1.438	0.284	0.066	(0.977, 2.117)
Constant	0.047	0.030	0.000	(0.013, 0.167)

**Table A3:** Log-binomial regression models for sleep disturbance due to railways in relation to the distance to railways. All variables are in a same model.

Variable	PPR	SE	*p*-Value	(95% CI)
Distance to a railway (m)				
>100	1			
101–150	0.409	0.124	0.003	(0.225, 0.743)
151–200	0.347	0.175	0.036	(0.129, 0.932)
201–500	0.066	0.031	0.000	(0.026, 0.167)
501–1000	0.076	0.035	0.000	(0.031, 0.188)
>1000	0.027	0.016	0.000	(0.008, 0.087)
Age				
18–29	1			
30–39	1.010	0.569	0.986	(0.334, 3.049)
40–49	0.483	0.251	0.161	(0.174, 1.337)
50–59	0.771	0.399	0.616	(0.280, 2.128)
60–69	1.116	0.627	0.845	(0.371, 3.355)
70–79	1.482	0.947	0.538	(0.424, 5.188)
≥80	1.743	1.233	0.433	(0.435, 6.979)
Education				
No diploma	1			
High school	1.849	1.312	0.387	(0.460, 7.431)
CEGEP	1.871	1.390	0.399	(0.436, 8.032)
University degree	2.688	1.884	0.158	(0.680, 10.623)
Sex				
Men	1			
Women	0.879	0.237	0.634	(0.518, 1.492)
Constant	0.060	0.051	0.001	(0.011, 0.318)

**Table A4:** Distance to railways and proportions of sleep disturbance due to railways in the weighted study sample, adjusted for sensitivity to noise: regression model.

Variable	PPR	SE	*p*-Value	(95% CI)
Distance to a Railway (m)				
≤100	1			
101–150	0.420	0.212	0.09	(0.156, 1.131)
151–200	0.267	0.178	0.05	(0.072, 0.989)
201–500	0.027	0.028	<0.01	(0.003, 0.209)
501–1000	0.014	0.010	<0.01	(0.004, 0.054)
>1000	0.024	0.026	<0.01	(0.003, 0.192)
Sensitivity				
Not sensitive	1			
Sensitive	1.387	0.603	0.45	(0.591, 3.255)
Distance to railway × Sensitivity interaction	(base levels omitted)			
101–150—Sensitive	0.924	0.602	0.90	(0.257, 3.318)
151–200—Sensitive	1.688	1.597	0.58	(0.264, 10.788)
201–500—Sensitive	3.628	4.379	0.29	(0.340, 38.668)
501–1000—Sensitive	8.142	6.781	0.01	(1.591, 41.669)
Age				
18–29	1			
30–39	0.941	0.526	0.91	(0.314, 2.816)
40–49	0.428	0.225	0.11	(0.153, 1.199)
50–59	0.662	0.339	0.42	(0.242, 1.806)
60–69	0.972	0.550	0.96	(0.320, 2.948)
70–79	1.359	0.848	0.62	(0.400, 4.621)
≥80	1.813	1.296	0.41	(0.446, 7.361)
Education				
No diploma	1			
High school	1.840	1.302	0.39	(0.459, 7.367)
CEGEP	1.710	1.259	0.47	(0.404, 7.243)
University degree	2.352	1.701	0.24	(0.569, 9.712)
Sex				
Men	1			
Women	0.836	0.230	0.52	(0.488, 1.433)
Constant	0.0610225	0.0522719	<0.01	(0.011, 0.327)

**Table A5:** Distance to railways and proportions of sleep disturbance due to railways in the weighted study sample, adjusted for sensitivity to noise: marginal prediction by sensitivity.

Variable	Sensitive Proportion (95% CI)		Not Sensitive Proportion (95% CI)		All Proportion (95% CI)	
≤100	12.32	(6.21, 18.44)	8.89	(3.36, 14.41)	10.67	(6.87, 14.46)
101–150	4.78	(2.06, 7.50)	3.73	(0.78, 6.68)	4.28	(2.29, 6.27)
151–200	5.56	(−1.40, 12.52)	2.37	(−0.40, 5.14)	4.02	(0.08, 7.97)
201–500	1.19	(0, 2.39)	0.24	(−0.23, 0.70)	0.73	(0.10, 1.37)
501–1000	1.44	(0.17, 2.70)	0.13	(−0.02, 0.28)	0.8	(0.16, 1.45)
>1000	0.35	(−0.14, 0.84)	0.21	(−0.21, 0.64)	0.28	(−0.04, 0.61)

**Table A6:** Distance to the NEF25 contour and marginal proportions of sleep disturbance by airplanes in the weighted study sample adjusted for sensitivity to noise: regression model.

Variable	PPR	SE	*p*-Value	(95% CI)
Distance to NEF25 contour (m)				
within NEF25	1			
1–1000	0.323	0.135	0.01	(0.142, 0.733)
1001–2000	0.126	0.090	0.00	(0.031, 0.512)
>2000	0.023	0.014	0.00	(0.006, 0.079)
Sensitivity				
Not sensitive	1			
Sensitive	0.975	0.438	0.95	(0.404, 2.350)
Distance to NEF25 × Sensitivity interaction	(base levels omitted)			
1–1000—Sensitive	2.223	1.240	0.15	(0.744, 6.635)
1001–2000—Sensitive	3.815	4.127	0.22	(0.458, 31.806)
>2000—Sensitive	3.233	2.548	0.14	(0.689, 15.161)
Age				
18–29	1			
30–39	7.240	7.884	0.07	(0.856, 61.224)
40–49	8.793	8.229	0.02	(1.404, 55.079)
50–59	7.167	6.388	0.03	(1.249, 41.133)
60–69	10.149	9.249	0.01	(1.700, 60.581)
70–79	4.807	4.428	0.09	(0.790, 29.255)
≥80	4.765	5.251	0.16	(0.549, 41.337)
Education				
No diploma	1			
High school	3.443	2.447	0.08	(0.854, 13.873)
CEGEP	2.323	1.574	0.21	(0.616, 8.768)
University degree	1.591	0.996	0.46	(0.467, 5.427)
Sex				
Men	1			
Women	1.204	0.385	0.56	(0.644, 2.252)
Constant	0.011	0.013	0.00	(0.001, 0.100)

**Table A7:** Distance to the NEF25 contour and marginal proportions of sleep disturbance by airplanes in the weighted study sample adjusted for sensitivity to noise: marginal prediction by sensitivity.

Variable	Sensitive Proportion (95% CI)		Not Sensitive Proportion (95% CI)		All Proportion (95% CI)	
Within NEF25	15.25	(7.15, 23.35)	15.57	(7.78, 23.37)	15.4	(9.61, 21.19)
1–1000	11.57	(8.38, 14.75)	5.80	(2.72, 8.88)	8.92	(6.69, 11.14)
1001–2000	8.15	(−2.30, 18.60)	2.37	(−0.53, 5.27)	5.49	(−0.23, 11.21)
>2000	1.36	(0.69, 2.04)	0.44	(−0.06, 0.94)	0.94	(0.51, 1.36)

**Table A8:** Distance to major roads proportions of sleep disturbance due to road traffic in the weighted study sample, adjusted for sensitivity to noise: regression model.

Variable	PPR1	SE	*p*-Value	(95% CI)
Distance to a major road (m)				
≤50	1			
51–100	−0.611	0.583	0.29	(−1.754, 0.531)
101–150	−0.455	0.723	0.53	(−1.873, 0.962)
151–200	−0.643	0.757	0.40	(−2.127, 0.841)
201–500	0.289	0.512	0.57	(−0.714, 1.293)
>500	−2.968	0.808	0.00	(−4.552, 1.383)
Sensitivity				
Not sensitive	1			
Sensitive	1.566	0.410	0.00	(0.763, 2.369)
Distance road × Sensitivity interaction	(base levels omitted)			
51–100—Sensitive	0.201	0.646	0.76	(−1.066, 1.468)
101–150—Sensitive	−0.356	0.826	0.67	(−1.976, 1.264)
151–200—Sensitive	0.021	0.877	0.98	(−1.698, 1.740)
201–500—Sensitive	−1.255	0.583	0.03	(−2.398, 0.112)
>500—Sensitive	1.369	0.980	0.16	(−0.552, 3.289)
Age				
18–29	1			
30–39	0.461	0.551	0.40	(−0.62, 1.541)
40–49	0.482	0.530	0.36	(−0.557, 1.521)
50–59	0.800	0.521	0.13	(−0.223, 1.822)
60–69	0.381	0.571	0.50	(−0.737, 1.500)
70–79	−0.132	0.666	0.84	(−1.438, 1.174)
≥80	0.470	0.762	0.54	(−1.025, 1.964)
Education				
No diploma	1			
High school	−1.023	0.453	0.02	(−1.912, 0.135)
CEGEP	−0.419	0.413	0.31	(−1.229, 0.390)
University degree	−0.665	0.392	0.09	(−1.434, 0.103)
Sex				
Men	1			
Women	0.308	0.199	0.12	(−0.082, 0.698)
Constant	−3.785	0.711	0.00	(−5.18, 2.391)

**Table A9:** Distance to major roads proportions of sleep disturbance due to road traffic in the weighted study sample, adjusted for sensitivity to noise: marginal prediction by sensitivity.

Variable	Sensitive Proportion (95% CI)		Not Sensitive Proportion (95% CI)		All Proportion (95% CI)	
≤50	11.62	(8.14, 15.10)	2.43	(0.61, 4.24)	7.29	(5.18, 9.39)
51–100	7.71	(4.15, 11.27)	1.32	(0.15, 2.48)	4.70	(2.86, 6.54)
101–150	5.16	(1.59, 8.74)	1.54	(−0.34, 3.42)	3.45	(1.40, 5.51)
151–200	6.24	(1.10, 11.37)	1.28	(−0.37, 2.92)	3.90	(1.13, 6.67)
201–500	4.43	(2.31, 6.54)	3.24	(1.04, 5.45)	3.87	(2.35, 5.38)
>500	2.35	(−0.13, 4.83)	0.12	(−0.05, 0.30)	1.30	(−0.01, 2.61)

**Table A10:** L_Night_ and marginal proportions of sleep disturbance by total environmental noise in the weighted study sample, adjusted for sensitivity to noise: regression model.

Variable	PPR	SE	*p*-Value	(95% CI)
L_Night_	0.988	0.021	0.57	(0.948, 1.03)
Sensitivity				
Not sensitive	1			
Sensitive	0.106	0.158	0.13	(0.006, 1.990)
L_night_ × Sensitive interaction	1.052	0.027	0.05	(1.000, 1.108)
Age				
18–29	1			
30–39	1.321	0.331	0.27	(0.808, 2.159)
40–49	1.356	0.320	0.20	(0.854, 2.154)
50–59	1.523	0.346	0.06	(0.975, 2.378)
60–69	1.322	0.323	0.25	(0.819, 2.134)
70–79	1.107	0.315	0.72	(0.634, 1.932)
≥80	1.236	0.439	0.55	(0.616, 2.481)
Education				
No diploma	1			
High school	0.732	0.191	0.23	(0.438, 1.222)
CEGEP	1.147	0.274	0.57	(0.718, 1.831)
University degree	0.973	0.223	0.90	(0.620, 1.526)
Sex				
Men	1			
Women	0.884	0.092	0.24	(0.721, 1.085)
Constant	0.139	0.170	0.11	(0.013, 1.532)

**Table A11:** L_Night_ and marginal proportions of sleep disturbance by total environmental noise in the weighted study sample, adjusted for sensitivity to noise: marginal prediction by sensitivity.

Variable	Sensitive Proportion (95% CI)		Not Sensitive Proportion (95% CI)		All Proportion (95% CI)	
45 dBA	11.59	(7.77, 15.41)	6.81	(4.32, 9.30)	9.36	(6.27, 12.45)
50 dBA	13.34	(10.43, 16.26)	7.39	(5.41, 9.36)	10.57	(8.30, 12.83)
55 dBA	15.36	(13.26, 17.46)	8.02	(6.40, 9.63)	11.94	(10.51, 13.37)
60 dBA	17.68	(15.08, 20.28)	8.70	(6.95, 10.45)	13.50	(11.87, 15.13)
65 dBA	20.36	(15.52, 25.20)	9.44	(6.91, 11.97)	15.27	(11.97, 18.57)
70 dBA	23.44	(15.20, 31.68)	10.24	(6.49, 14.00)	17.29	(11.54, 23.04)

**Table A12:** L_Night_ and marginal proportions of sleep disturbance by transportation noise in the weighted study sample, adjusted for sensitivity to noise: regression model.

Variable	PPR	SE	*p*-Value	(95% CI)
L_Night_	0.969	0.031	0.32	(0.91, 1.031)
Sensitivity				
Not sensitive	1			
Sensitive	0.009	0.020	0.03	(0, 0.691)
Lnight × Sensitive interaction	1.105	0.043	0.01	(1.024, 1.192)
Age				
18–29	1			
30–39	1.554	0.611	0.26	(0.719, 3.359)
40–49	1.484	0.556	0.29	(0.712, 3.094)
50–59	1.825	0.674	0.10	(0.885, 3.763)
60–69	1.687	0.660	0.18	(0.783, 3.634)
70–79	1.042	0.489	0.93	(0.415, 2.612)
≥80	1.505	0.833	0.46	(0.508, 4.457)
Education				
No diploma	1			
High school	0.614	0.225	0.18	(0.299, 1.261)
CEGEP	0.837	0.295	0.61	(0.419, 1.671)
University degree	0.721	0.239	0.33	(0.376, 1.383)
Sex				
Men	1			
Women	1.175	0.180	0.29	(0.87, 1.586)
Constant	0.162	0.311	0.34	(0.004, 6.979)

**Table A13:** L_Night_ and marginal proportions of sleep disturbance by transportation noise in the weighted study sample, adjusted for sensitivity to noise: marginal prediction by sensitivity.

Variable	Sensitive Proportion (95% CI)		Not Sensitive Proportion (95% CI)		All Proportion (95% CI)	
45 dBA	4.82	(2.38, 7.26)	0.96681	(0.97, 3.5)	3.60	(1.78, 5.42)
50 dBA	6.17	(4.09, 8.24)	1.52281	(1.52, 3.73)	4.50	(3.02, 5.98)
55 dBA	7.89	(6.26, 9.53)	2.10621	(2.11, 4.06)	5.63	(4.62, 6.64)
60 dBA	10.10	(7.98, 12.23)	2.50965	(2.51, 4.74)	7.05	(5.87, 8.24)
65 dBA	12.93	(8.5, 17.36)	2.56426	(2.56, 5.95)	8.85	(6.1, 11.6)
70 dBA	16.55	(8.07, 25.03)	2.30118	(2.3, 7.71)	11.12	(5.71, 16.52)

**Table A14:** L_Night_ and marginal proportions of sleep disturbance by road noise in the weighted study sample, adjusted for sensitivity to noise: regression.

Variable	PPR	SE	*p*-Value	(95% CI)
L_Night_	1.002	0.039	0.96	(0.928, 1.082)
Sensitivity				
Not sensitive	1			
Sensitive	0.006	0.015	0.05	(0, 0.918)
L_Night_ × Sensitive interaction	1.117	0.050	0.01	(1.022, 1.22)
Age				
18–29	1			
30–39	1.645	0.839	0.33	(0.606, 4.471)
40–49	1.734	0.875	0.28	(0.644, 4.666)
50–59	2.449	1.222	0.07	(0.921, 6.512)
60–69	1.609	0.878	0.38	(0.552, 4.687)
70–79	0.993	0.637	0.99	(0.282, 3.492)
≥80	1.867	1.343	0.39	(0.456, 7.65)
Education				
No diploma	1			
High school	0.343	0.153	0.02	(0.144, 0.82)
CEGEP	0.688	0.278	0.36	(0.312, 1.518)
University degree	0.563	0.213	0.13	(0.268, 1.183)
Sex				
Men	1			
Women	1.338	0.265	0.14	(0.907, 1.974)
Constant	0.015	0.036	0.08	(0, 1.745)

**Table A15:** L_Night_ and marginal proportions of sleep disturbance by road noise in the weighted study sample, adjusted for sensitivity to noise: marginal prediction by sensitivity.

Variable	Sensitive Proportion (95% CI)		Not Sensitive Proportion (95% CI)		All Proportion (95% CI)	
45 dBA	1.93	(0.82, 3.05)	0.25	(1.25, 2.15)	1.38	(0.59, 2.17)
50 dBA	3.12	(1.87, 4.37)	0.52	(1.66, 3)	2.17	(1.33, 3.01)
55 dBA	5.04	(3.73, 6.35)	0.89	(2.28, 4.21)	3.42	(2.63, 4.21)
60 dBA	8.13	(6.25, 10.01)	1.30	(3.3, 6.44)	5.41	(4.37, 6.44)
65 dBA	13.12	(8.61, 17.63)	1.63	(5.06, 11.23)	8.55	(5.86, 11.24)
70 dBA	21.18	(10.34, 32.02)	1.73	(8, 20.13)	13.55	(6.9, 20.2)

**Table A16:** Log-binomial regression models for sleep disturbance by transportation noise according to proximity to single and combined sources of transportation noise *.

Variable	PPR	SE	*p*-Value	(95% CI)
Not exposed	1			
Airplane only	3.440	0.589	<0.01	(2.459, 4.814)
Road only	1.852	0.348	<0.01	(1.281, 2.678)
Railway only	2.615	0.576	<0.01	(1.698, 4.026)
Road & Aircraft	2.406	0.656	<0.01	(1.409, 4.107)
Road & Railway	3.764	0.860	<0.01	(2.405, 5.890)
Age				
18–29	1			
30–39	7.240	7.884	0.07	(0.856, 61.224)
40–49	8.793	8.229	0.02	(1.404, 55.079)
50–59	7.167	6.388	0.03	(1.249, 41.133)
60–69	10.149	9.249	0.01	(1.700, 60.581)
70–79	4.807	4.428	0.09	(0.790, 29.255)
≥80	4.765	5.251	0.16	(0.549, 41.337)
Education				
No diploma	1			
High school	3.443	2.447	0.08	(0.854, 13.873)
CEGEP	2.323	1.574	0.21	(0.616, 8.768)
University degree	1.591	0.996	0.46	(0.467, 5.427)
Sex				
Men	1			
Women	1.204	0.385	0.56	(0.644, 2.252)
Constant	0.011	0.013	<0.01	(0.001, 0.100)

* Airplane (≤1000 m from NEF25 or in NEF25), Road (≤100 m from an artery or highway), Railway (≤150 m from a railway line or main line of a railroad shunting yard).
